# Novel Assay to Measure Seroprevalence of Zika Virus in the Philippines

**DOI:** 10.3201/eid2712.211150

**Published:** 2021-12

**Authors:** Cameron Adams, Ramesh Jadi, Bruno Segovia-Chumbez, Jedas Daag, Michelle Ylade, Freddy A. Medina, Tyler M. Sharp, Jorge L. Munoz-Jordan, In-Kyu Yoon, Jacqueline Deen, Anna Lena Lopez, Aravinda M. de Silva, Lakshmanane Premkumar

**Affiliations:** University of North Carolina School of Medicine, Chapel Hill, North Carolina, USA (C. Adams, R. Jadi, B. Segovia-Chumbez, A.M. de Silva, L. Premkumar);; University of the Philippines, Manila, Philippines (J. Daag, M. Ylade, J. Deen, A.L. Lopez);; Centers for Disease Control and Prevention, San Juan, Puerto Rico, USA (F.A. Medina, T.M. Sharp, J.L. Munoz-Jordan);; Coalition for Epidemic Preparedness Innovations, Washington, DC, USA (I.-K. Yoon)

**Keywords:** Zika virus, arboviruses, dengue virus, diagnostics, ELISA testing, flaviviruses, mosquito-borne diseases, neutralizing antibody tests, Philippines, serology, seroprevalence, vector-borne infections, viruses

## Abstract

Zika virus (ZIKV) is a member of the *Flaviviridae* family, which includes other clinically notable viruses such as the 4 dengue virus serotypes (DENV-1–4). Distinguishing DENVs from ZIKV using the established serologic assays widely used for monitoring DENV transmission is difficult because of antibody cross-reactivity between these closely related flaviviruses. We describe a modified and improved recombinant envelope domain III–based serologic assay for detecting ZIKV type-specific antibodies in regions with endemic DENV transmission. When the assay was used to measure ZIKV seroprevalence in 2017 among children 9–14 years of age living in a region of the Philippines with endemic DENV transmission, we observed a ZIKV seroprevalence of 18%. Investigators should consider using the ZIKV envelope domain III–based assay, which is simple and readily adaptable for use in standard clinical and public health laboratories, to assess ZIKV seroprevalence in areas with endemic DENV transmission.

Zika virus (ZIKV) is a positive sense RNA virus of the *Flaviviridae* family, which includes several medically notable arboviruses such as dengue (DENV), yellow fever, Japanese encephalitis virus, and West Nile virus. Before the 2015 epidemic in the Americas that spread to >40 countries and infected >1 million people (*1*), ZIKV was considered rare and responsible for minor epidemics in East Africa and parts of Asia. Although most ZIKV infections are asymptomatic or clinically mild, the epidemic in the Americas revealed that the virus can cause serious neurologic problems in some persons and severe teratogenic effects in pregnant women (*2*–*4*). Because ZIKV and DENV-1–4 share the same *Aedes* mosquito vectors, areas in the Americas most affected by ZIKV also experience endemic DENV transmission. The ZIKV pandemic in the Americas led to novel observations and questions about its epidemiology and pathogenesis in regions with endemic DENV transmission. Recent studies indicate that cross-reactive immunity between ZIKV and DENV can lead to protection or to more severe disease depending on the context (*5*–*7*).

Although sporadic transmission of Asian lineages of ZIKV in Southeast Asia and Pacific islands is well-documented (*8*,*9*), its prevalence in the region has been difficult to estimate using current serologic assays because of intense transmission of multiple DENV serotypes and antibody cross-reactivity between DENV and ZIKV. Most serologic assays for flaviviruses measure antibodies binding to viral-envelope glycoprotein (E protein) because this antigen is a major target of human antibodies. The *Flavivirus* E protein contains immunodominant antibody epitopes that are conserved (cross-reactive) between different flaviviruses or unique to each virus (type-specific) (*10*–*12*). 

Traditional *Flavivirus* serologic assays exhibit poor specificity in distinguishing DENV from ZIKV infections because these assays use whole virions or E proteins containing conserved epitopes as antigens (*13*–*15*). More recently, the ZIKV epidemic in the Americas spurred the development of recombinant viral antigens and serologic assays for distinguishing ZIKV from DENV (*16*–*18*). We previously described a serologic assay using domain III of the ZIKV E protein (EDIII) to detect ZIKV type-specific antibodies among persons in areas with DENV and ZIKV cocirculation (*19*). Here we describe development of a second-generation ZIKV EDIII–based serologic assay and its use to measure the seroprevalence of ZIKV among children 9–14 years of age in the Cebu Province of the Philippines. 

## Materials and Methods

### ZIKV EDIII Antigen Production

We expressed a codon-optimized gene encoding for EDIII from ZIKV strain H/PF/2013 in Expi293 cells as a fusion protein containing a human serum albumin signal peptide for secretion, a polyhistidine tag (his-tag) for affinity purification, and a HaloTag (Promega, https://www.promega.com) for biotinylation (*20*). We deposited the nucleotide sequence of the construct into Genbank (accession no. MZ592925). HaloTag enables single, site-specific biotinylation distant from the folded EDIII protein. We purified recombinant EDIII antigen from the culture supernatant using nickel-nitrilotriacetic acid agarose (QIAGEN, https://www.qiagen.com) and biotinylated it using HaloTag PEG biotin ligand (Promega), according to manufacturer protocol. We analyzed the identity and purity of the biotinylated EDIII antigen using SDS-PAGE (sodium dodecyl-sulfate polyacrylamide gel electrophoresis) mobility-shift analysis.

### ZIKV EDIII ELISA

We coated a 96-well high binding microtiter plate (Greiner Bio-One, https://www.gbo.com) with 50 μL of streptavidin at 4 μg/mL in tris-buffered saline (TBS, pH 7.4) for 1 h at 37°C. We captured the biotinylated EDIII at 2 μg/mL in TBS, washed the plate 3 times with wash buffer (TBS containing 0.2% Tween 20), and then blocked it with 100 μL of blocking solution (3% milk in TBS containing 0.05% Tween 20) for 1 h at 37°C. After removing the blocking solution, we added 50 μL of heat-inactivated (56°C for 30 min) serum sample at 1:20 or indicated dilutions in blocking buffer and incubated for 1 hour at 37°C. After washing the plate in the wash buffer, we added 50 μL of alkaline phosphatase-conjugated secondary goat anti-human secondary IgG (Sigma) at 1:2,500 dilution for 1 hour at 37°C. We washed the plate, added 50 μL of p-nitrophenyl phosphate substrate (Sigma, https://www.sigmaaldrich.com), and measured absorbance at 405 nm using an Epoch plate reader (Biotek, https://www.biotek.com). For analyzing the 547 serum samples from the Cebu cohort using ELISA assays performed over several days, we used human monoclonal antibody ZKA190 (*21*), which binds to highly accessible regions of ZIKV EDIII as a control to standardize EDIII ELISA optical density (OD) values across assays. Each plate was developed until wells with ZKA190 generated an OD value within a 1.0–1.3 range. If the signal was outside this range, we considered the assay invalid and repeated the process. We divided all OD values for human samples by the ZKA190 OD value on the same plate before determining the ZIKV-immune status of each participant (>0.34 cutoff).

### Human Serum Panel Used to Validate the EDIII Assay

To validate the EDIII assay as described elsewhere (*19*), we used a panel of 142 archived convalescent serum samples from 15 participants who received a licensed *Flavivirus* (yellow fever/Japanese encephalitis virus) vaccine, 27 serum samples from *Flavivirus*-naive participants, 33 from participants with immunity to ZIKV (including some with both ZIKV and DENV immunity), and 67 from participants with DENV immunity but no immunity to ZIKV. The DENV- and ZIKV-immunity status of convalescent specimens in the panel was based on participants in febrile illness study cohorts in Nicaragua and Sri Lanka with laboratory-confirmed acute DENV or ZIKV infections or tests for the presence of neutralizing DENV or ZIKV antibodies in single serum samples from healthy persons. We designated samples with no detectable neutralizing DENV and ZIKV antibodies or 50% plaque reduction neutralization test (PRNT_50_) results <10 as naive. Collection, storage, and use of these convalescent serum samples for research was approved by the Institutional Review Board of the University of North Carolina at Chapel Hill (protocol 08–0895). 

### Centers for Disease Control and Prevention ZIKV Persistence Samples

The Centers for Disease Control and Prevention (CDC) and the Ponce Medical School Foundation monitored DENV and ZIKV patients seeking treatment at 2 hospitals in southern Puerto Rico. During triage, we identified case-patients with >1 signs or symptoms: fever (temperature >38.0°C or >100.5°F) or reporting fever lasting <7 days, rash, arthralgia or arthritis, or conjunctivitis and offered them participation in the study. We identified ZIKV cases through test results from the CDC Dengue Branch laboratory in San Juan, Puerto Rico, where reverse transcription (RT) PCR testing for DENV, ZIKV, CHIKV, and other respiratory infectious diseases is performed and followed ZIKV cases longitudinally as described elsewhere (*22*). For our study, we selected and prospectively followed 27 ZIKV RT-PCR–positive cases for up to 2.5 years. Specimen collection was approved by institutional review boards at CDC and Ponce Medical School Foundation.

### Pediatric Samples from Cebu Province

We published the Cebu study protocol and approvals elsewhere (*23*). In brief, our study used baseline serum samples that were collected from 2,996 children 9–14 years of age enrolled in a postlicensure DENV vaccine study. Cohort residence was equally split between Bogo and Balamban, both semiurban areas. We collected samples and demographic information before participants were vaccinated at the same visit. 

### DENV and ZIKV Focus Reduction Neutralization Test

We determined neutralization titers against DENV and ZIKV by focus-reduction neutralization test (FRNT) in a 96-well format described elsewhere (*24*). Serially diluted serum was mixed with 50–100 focus-forming units of the virus in Dulbecco modified Eagle medium with 2% fetal bovine serum. We incubated the antibody and virus complexes (1 h, 37°C), then transferred them to a monolayer of Vero-81 cells for infection. After 1 additional hour of incubating antibody and virus complex on Vero-81 monolayer, we overlaid cells with GIBCO Opti-MEM (https://www.thermofisher.com), a modified Eagle medium containing 2% fetal bovine serum and 1% carboxymethylcellulose. After allowing the predetermined time required to form viral foci, we fixed Vero-81 cells and immunostained them with *Flavivirus*-specific monoclonal antibodies. For neutralization assays, we calculated 50% inhibitory concentration by using the sigmoidal dose-response (variable slope) equation in Prism 6 (GraphPad Software, https://www.graphpad.com). For the study, we included only reported values with an R^2^ (coefficient of determination) >0.75, hill slope >0.5, and 50% inhibitory concentration within the assay range. We performed FRNT for *Flavivirus* strains WP74 (DENV-1), S16803 (DENV-2), CH53489 (DENV-3), TVP-376 (DENV-4), and H/PF/2013 (ZIKV).

### Depletion of DENV binding Antibodies

We obtained purified viral antigens for antibody depletions by infecting Vero-81 cell cultures in 850 cm^2^ roller bottles (Greiner Bio-One, https://www.gbo.com) as described elsewhere (*25*). We conjugated *Flavivirus-*specific antibody 1M7 to Tosyl-activated Dynabeads (Thermo Fisher) magnetic beads and incubated purified DENV-1–4 viral antigens (1 h at 37°C) with the beads. To deplete DENV-specific antibodies, we incubated serum samples (3 h at 37°C) with DENV-conjugated Dynabeads and incubated serum samples with Dynabeads conjugated to an equivalent bovine serum albumin concentration. We confirmed cross-reactive and DENV antibody depletion using whole virion capture ELISA against DENV-1–4. We measured serotype-specific ZIKV antibodies in serum samples using ZIKV whole-virion capture ELISA after depletion, as described elsewhere (*24*).

### Receiver Operator Characteristic Analysis

We used SPSS Statistics for Macintosh version 27.0 (https://www.ibm.com) to report the performance of the EDIII ELISA based on the receiver operating characteristic (ROC) curve, which presents test performance as true-positive (sensitivity) versus false-positive (1 – specificity). We calculated the optimal assay cutoff value, which maximizes sensitivity and specificity, from the ROC curve using the same software. According to the test, sensitivity is the fraction of total confirmed positive samples with true positives, and specificity is total confirmed negative samples with true negatives. 

## Results

### Development of Immunoassay for Detecting ZIKV Antibodies in Patient Serum

**Figure 1:**
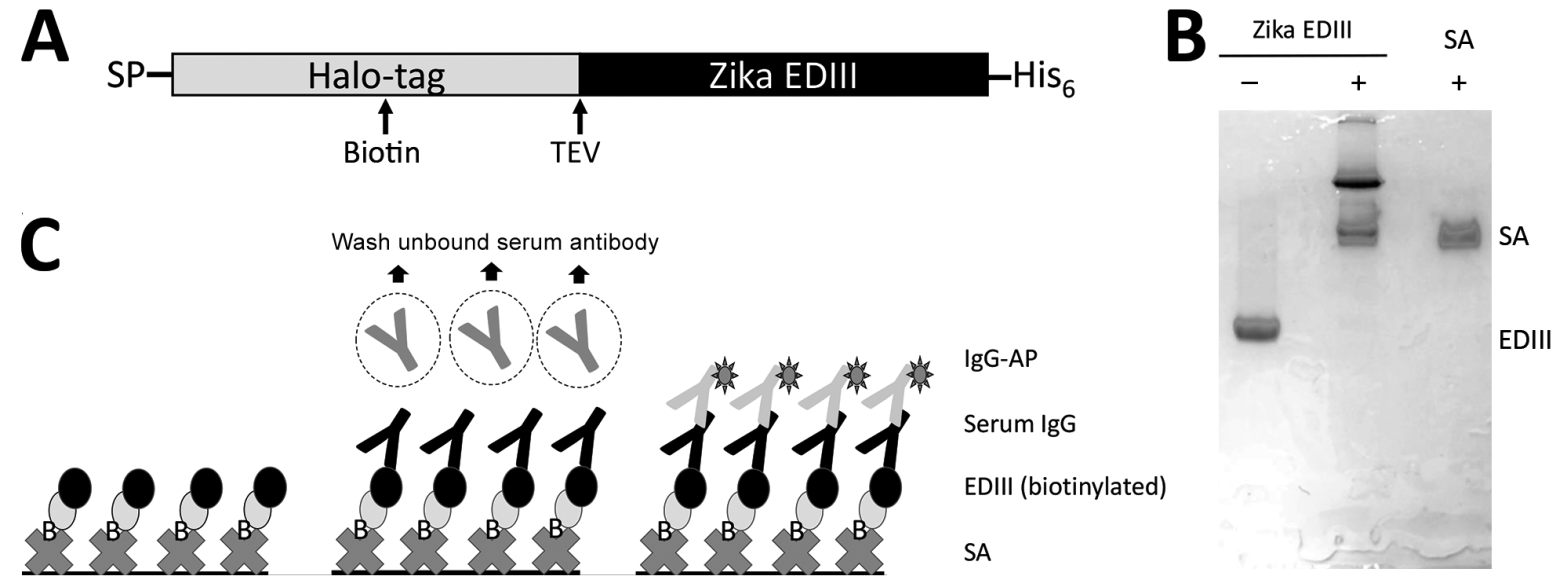
ZIKV Biotinylated-EDIII antigen capture ELISA in study of novel assay to measure ZIKV seroprevalence in the Philippines. A) Schematic of the ZIKV EDIII construct with an N-terminal human albumin secretion signal, a HaloTag (Promega, https://www.promega.com) for site-specific biotinylation and a C-terminal 6-histidine residue tag for affinity purification. The EDIII construct design also included a TEV protease cleavage site between HaloTag and EDIII. B) Biotinylated ZIKV EDIII displays an electrophoretic mobility shift with streptavidin. A site-specific biotinylated ZIKV EDIII was prepared using HaloTag biotin ligand. Electrophoretic gel shift analysis was performed in sodium dodecyl sulphate–polyacrylamide gel electrophoresis with biotinylated EDIII antigen in the presence and absence of streptavidin. C) Schematic of second-generation ZIKV EDIII ELISA using streptavidin-biotin interaction. Biotinylated EDIII antigen is captured by plate immobilized streptavidin. The antibody bound to EDIII is detected by a secondary anti–human IgG conjugated to alkaline phosphatase. EDIII, E protein domain III; IgG-AP, secondary IgG antibody conjugated to alkaline phosphatase; SA, streptavidin; SP, N terminal human albumin secretion signal; TEV, tobacco etch virus protease cleavage site; ZIKV, Zika virus.

We previously demonstrated that a serologic assay using the ZIKV EDIII fused to maltose-binding protein (MBP) reliably differentiated persons with past ZIKV infections from those with DENV infections (*19*). However, we observed a high background signal in some specimens, originating from human antibodies binding to MBP or the mouse antigen capture antibody used in the assay. To overcome this problem in the earlier version of the assay, we produced the ZIKV EDIII antigen fused to a HaloTag (Figure 1, panel A), derived from the haloalkane dehalogenase enzyme from *Rhodococcus rhodochrous* bacteria (*26*). By using a biotin HaloTag ligand (*27*), we added a single biotin molecule for each protein molecule at a site distant from the EDIII antigen (Figure 1, panel B). Using streptavidin-coated ELISA plates to capture the antigen, we established an assay for detecting ZIKV EDIII–binding antibodies in human clinical samples (Figure 1, panel C).

### Performance of the Second-Generation ZIKV EDIII ELISA

**Figure 2:**
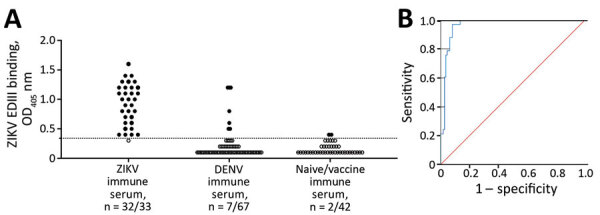
Performance evaluation of ZIKV EDIII assay in study of novel assay to measure ZIKV seroprevalence in the Philippines. Shown are the binding (A) and ROC (B) curve analysis of ZIKV EDIII ELISA using human convalescent serum samples. A panel of convalescent serum samples collected >12 weeks after onset of symptoms from primary and secondary ZIKV infections (n = 33), primary and multitypic DENV infections (n = 67), and serum samples collected >12 weeks after vaccination with a licensed *Flavivirus* vaccine or serum samples from *Flavivirus*-naive participants (n = 42) were tested by ZIKV EDIII ELISA. ROC demonstrated 0.966 (95% CI 0.94–0.99) area under the curve. The sensitivity of the EDIII capture ELISA was 97% (32/33) and the specificity 92% (100/109) at a cutoff value of 0.34. Red line indicates a random classifier and represents data points with equal true-positive rate and false-positive rate. The blue line is the ROC curve showing high performance of the ZIKV EDIII assay because the blue line is above and further away from a random classifier. DENV, dengue virus; EDIII, E protein domain III; OD_405_, optical density at 405 nm; ROC, receiver operating characteristic; ZIKV, Zika virus.

We performed an ROC analysis to determine the diagnostic performance of the new EDIII-capture ELISA in a panel of serum samples collected >12 weeks after symptom onset among persons with laboratory-confirmed ZIKV or DENV infections or who received a licensed *Flavivirus* vaccine. The panel included serum samples from DENV-naive and DENV-immune participants who received positive ZIKV RT-PCR test results, archived serum samples from persons who had primary (only 1 serotype) or secondary (>2 serotypes) DENV infections, control samples from persons who had not experienced DENV or ZIKV infections, and samples from travelers who had received Japanese encephalitis virus vaccine or yellow fever virus vaccine or both. The EDIII assay was positive for 32 of 33 ZIKV-immune, 7 of 67 DENV-immune, and 2 of 42 DENV- and ZIKV-naive or yellow fever/Japanese encephalitis virus vaccine recipients (Figure 2, panel A). The ROC analysis demonstrated an area under the curve of 0.97 (95% CI of 0.94–0.99) (Figure 2, panel B). At a cutoff value of OD 0.34, the sensitivity of the EDIII-capture ELISA was 97% and the specificity was 92%.

### Durability of ZIKV EDIII Antibodies in Persons Exposed to ZIKV Infections

**Figure 3:**
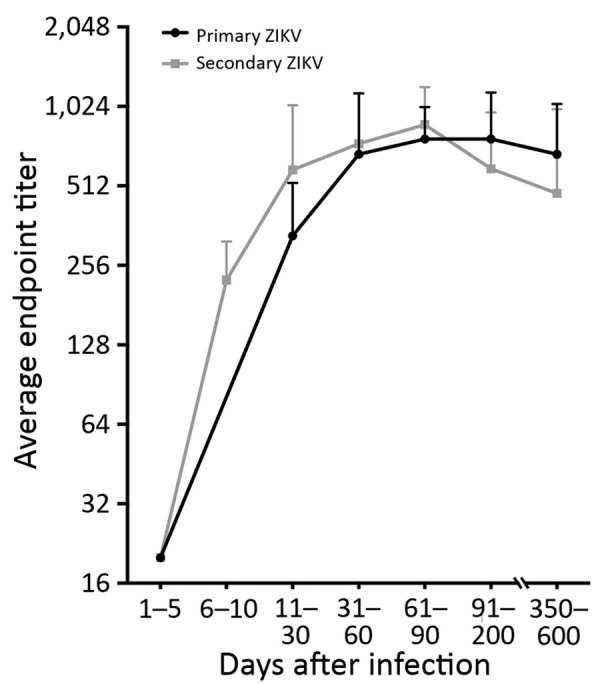
Durability of ZIKV EDIII antibodies in study of novel assay to measure ZIKV seroprevalence in the Philippines. Longitudinal samples from patients with PCR-confirmed ZIKV infections were collected 1–600 days after symptom onset and tested for ZIKV EDIII–binding antibodies. DENV serostatus was determined by DENV focus reduction neutralization test. Primary ZIKV serostatus indicates ZIKV infection in DENV-naive participants; secondary ZIKV serostatus indicates ZIKV infection in participants previously infected with DENV. DENV, dengue virus; EDIII, E protein domain III; ZIKV, Zika virus.

Next, we assessed the durability of EDIII antibodies using a panel of 98 longitudinal samples collected 1–600 days after symptom onset from 27 residents of Puerto Rico with PCR-confirmed ZIKV infections. By testing the acute specimens for DENV-specific antibodies, we stratified the DENV-immune status at the time of ZIKV infection as 12 DENV-naive and 15 DENV-immune cases. We measured endpoint titers at each time point to follow the kinetics of serum ZIKV EDIII antibodies (Figure 3). The ZIKV EDIII antibody titers reached peak levels by 1 month after initial infection and stayed well above detection level for ≥20 months in DENV- naive and DENV-immune participants.

### Seroprevalence of ZIKV in Cebu Province

**Figure 4:**
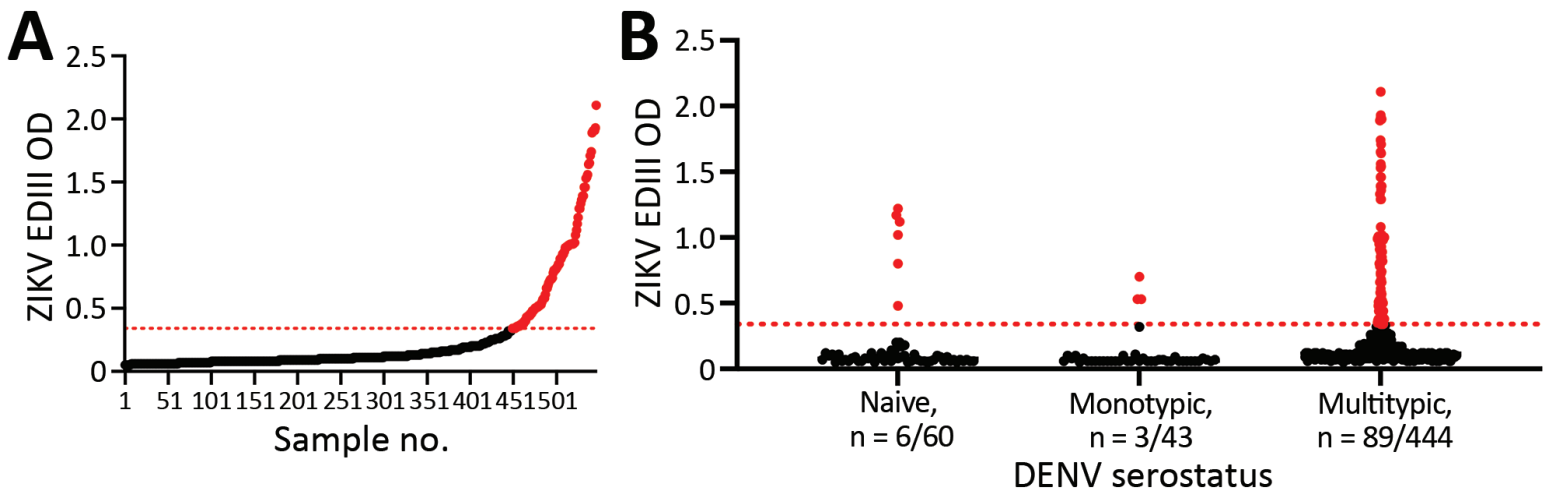
ZIKV positivity in Cebu, Philippines, in study of novel assay to measure ZIKV seroprevalence in the Philippines. A) Distribution of ZIKV EDIII antibody reactivity among the 547 participants tested; 18% of the participants tested positive. B) Distribution of ZIKV EDIII–positive participants by DENV serostatus. Horizontal dotted red lines indicate positive threshold of EDIII assay. DENV, dengue virus; EDIII, E protein domain III; OD, optical density; ZIKV, Zika virus.

Having established the performance of the ZIKV EDIII ELISA among participants exposed to multiple DENV serotypes, we used the assay to estimate the seroprevalence of ZIKV among children living in Balamban and Bogo City in Cebu Province. In 2017, we collected baseline blood samples from a DENV vaccine study of 2,996 children 9–14 years of age. Elsewhere we reported that 89.3% of the children were DENV-immune at baseline, demonstrating the high endemicity of the virus in this population (*23*). We selected a representative sample of 547 children on the basis of DENV-immune status from the baseline cohort to determine the seroprevalence of ZIKV (Table). The children selected for the study consisted of 60 (11%) with no immunity to DENV, 43 (8%) with immunity to 1 DENV serotype, and 444 (81%) with immunity to >2 serotypes (Table; Figure 4, panel B) (*23*). We observed that 98/547 (18%) children were ZIKV antibody positive in the ZIKV EDIII assay (Figure 4, panel A). The number of children who tested positive or negative for ZIKV antibodies did not differ by sex, location, or prior DENV-immune status (Table).

**Table:** Demographics of study cohort and number of participants with ZIKV EDIII type-specific antibody in study of novel assay to measure ZIKV seroprevalence in the Philippines

**Category**	**Total no. (%)**	**No. (%) ZIKV positive **	p value*
All participants	547	98 (18)	
Sex			0.50
M	218 (40)	35 (16)	
F	329 (60)	63 (19)	
Location			0.90
Balamban	145 (27)	25 (17)	
Bogo	402 (73)	73 (18)	
Specific type			0.06
Naive	60 (11)	6 (10)	
Monotypic	43 (8)	3 (7)	
Multitypic	444 (81)	89 (20)	

*Association between ZIKV serostatus and sex or location measured by Fisher exact test. Association between ZIKV serostatus and DENV-type serostatus measured by χ^2^ test.

### Comparison of ZIKV EDIII ELISA and Virus Neutralizing Antibody Assays

The current standard for estimating the seroprevalence of a particular flavivirus is based on measuring virus neutralizing antibodies in cell culture systems. Although neutralizing antibody assays are more specific than assays that measure antibody binding to flaviviruses or whole recombinant proteins, persons exposed to multiple DENV serotypes can develop ZIKV cross-neutralizing antibodies as reported elsewhere (*10*,*24*,*28*,*29*). Given the large number of children in our cohort with a history of >2 DENV serotype infections, we compared the performance for estimating ZIKV seroprevalence of the EDIII ELISA using FRNT.

From the 547 baseline samples tested by ZIKV EDIII assay, we tested 495 samples in a single dilution (1:40) ZIKV neutralization assay. We have demonstrated elsewhere that at 1:40 dilution, neutralization of >70% of the input virus is a reliable screening criterion for previous DENV infection (*23*). When we used the same criterion for the single-dilution ZIKV neutralization assay, we observed a much higher seroprevalence (39%) compared with the estimate (18%) based on the EDIII ELISA (Appendix Table 1). Most of the discordant samples (positive in the ZIKV neutralization assay and negative in the ZIKV EDIII assay) were from children with preexisting multitypic immunity to DENVs (105/110 participants) (Appendix Table 1), raising the possibility that high levels of DENV antibodies cross-neutralize ZIKV.

To evaluate whether the discordant results were because of the poor specificity of the ZIKV neutralization assay or poor sensitivity of the ZIKV EDIII ELISA, we selected 24 samples that had been classified by full curve neutralization testing against the 4 DENV serotypes and ZIKV as primary DENV-immune only (n = 3), primary ZIKV-immune only (n = 4), both primary DENV- and primary ZIKV-immune (n = 6), multitypic DENV-immune only (n = 2), or both multitypic DENV- and ZIKV-immune (n = 9) participants (Appendix Table 2, Figure 2) for further study. By selectively removing all antibodies to DENVs in a sample before testing for ZIKV binding antibodies, it is possible to detect ZIKV type-specific antibodies indicative of a past ZIKV infection (*11*). We incubated all 24 samples with magnetic beads coated with the 4 DENV serotypes to remove all DENV-binding antibodies. After confirming removal of all DENV-binding antibodies, we tested the samples for ZIKV-binding antibodies. After depleting DENV-binding antibodies, we observed ZIKV-binding antibodies for the participants designated as immune to primary Zika only and primary DENV and primary ZIKV (Appendix Table 2, Figure 1, panel A). In contrast, 6/9 participants designated by neutralization testing as multitypic DENV- and ZIKV-immune showed no ZIKV-binding antibodies (Appendix Figure 1, panel A). All participants that retained ZIKV-binding antibodies after DENV-binding antibody depletion also tested positive in the ZIKV EDIII ELISA and all participants with no detectable ZIKV-specific antibodies tested negative in the EDIII assay (Appendix Table 2, Figure 1, panel B). These results suggest that the ZIKV EDIII ELISA is more reliable than neutralization testing for estimating ZIKV seroprevalence independent of DENV status.

## Discussion

The 2015 ZIKV pandemic in the Americas mostly affected communities with high DENV endemicity because these viruses share the same mosquito vector. Efforts to monitor the spread of ZIKV and the effect of cross-reactive immunity on viral pathogenesis were severely hampered by the inability of conventional serologic assays to accurately distinguish DENV from ZIKV seropositivity. Several groups developed ZIKV recombinant antigen–based assays or antigen-antibody competition assays to improve the specificity of serologic assays (*16*–*18*).

In a previous study, we documented development of an ELISA to detect antibodies in persons who had recovered from ZIKV infections, using ZIKV EDIII fused to *Escherichia coli* MBP as an antigen (*19*). We observed in some persons high background levels of antibodies to the MBP fusion protein alone, most likely from natural exposure to bacterial proteins, highlighting the need for modifying the fusion protein used for antigen production. We describe a ZIKV EDIII antigen improved by replacing the MBP fusion tag with a HaloTag amenable to site-specific biotinylation. Using streptavidin-biotin chemistry to capture the antigen, we developed an ELISA with 97% sensitivity and 92% specificity for detecting past ZIKV infections.

A strength of our study was the use of serum samples from participants living in *Flavivirus*-endemic regions with well-defined exposures to DENVs or ZIKV or both to determine the performance of the assay when testing specimens with high levels of cross-reactive antibodies. A potential problem with using modular domains such as EDIII instead of the full-length envelope protein is a loss in assay sensitivity over time. However, our analysis of the longitudinal samples from patients with documented ZIKV infection demonstrated that ZIKV EDIII antibodies robustly developed and remained at high levels even after 2.7 years. This finding suggests that serologic assays based on EDIII will have the necessary sensitivity to characterize past ZIKV circulation at individual and population levels.

The severity of the epidemic in the Americas renewed interest in the epidemiology and pathogenesis of ZIKV in Asia. Asian lineages of the virus have been circulating for decades if not longer. The virus has been detected by molecular methods or virus isolation by cell culture in many countries, including Thailand, the Philippines, Vietnam, Malaysia, Cambodia, and India (*30*–*35*). One study of 600 migrant workers in Taiwan (predominantly from Indonesia, the Philippines, Thailand, and Vietnam) reported 37% seroprevalence using a ZIKV IgG assay (*36*,*37*). A similar study of Taiwan residents (n = 212) identified 4.2% of all participants with ZIKV IgG but confirmed only 1 participant by neutralization test (*36*). Investigators tested a cohort of children 1–4 years of age (n = 662) in Indonesia and estimated 9.1% seroprevalence using 90% neutralization as a cutoff value (*38*). A 2017 study of healthy adults (n = 801) in Vietnam estimated 1.1% seroprevalence using a ZIKV neutralization assay (*39*). An additional study in Guangxi Province, China, in March 2019 found 6% of 273 participants bound to ZIKV NS1 using IgG ELISA testing and neutralized ZIKV in cell cultures (*40*). Those past studies, especially those relying on tests of whole Zika virions and full-length antigens, had poor specificity, mainly because of antibodies induced by DENV infections cross-reacting with ZIKV antigens. In our study, we used a recombinant ZIKV antigen with a high specificity for distinguishing ZIKV-induced from DENV-induced antibodies; results showed that 18% of children 9–14 years of age living in Cebu Province had previously experienced a ZIKV infection. Although ZIKV is currently not considered by public health agencies to be a common infection in the Philippines, our results indicate otherwise.

Our results also highlight a problem with using accepted standards for neutralizing antibody testing to identify ZIKV infections in populations heavily exposed to DENVs. We observed ZIKV neutralizing antibodies in 39% of the children in this study, which was more than double the estimate based on the EDIII assay results. By depleting all DENV-binding antibodies from a subset of samples and then measuring ZIKV antibodies, we demonstrated that some children exposed to >2 DENV serotypes have low levels of ZIKV cross-neutralizing antibodies that can lead to false-positive results. In contrast, the ZIKV EDIII assay was not impacted by DENV-immune status because, even after removing all DENV-binding antibodies, the children maintained ZIKV EDIII–binding antibodies.

The novel assay we describe, which uses a biotinylated EDIII antigen to detect ZIKV type-specific antibodies, is simple and readily adaptable for use in standard clinical and public health laboratories to monitor ZIKV transmission at both the individual and population levels. Given that the 2015 ZIKV epidemic spread to >40 countries and infected more than a million people, many in regions with endemic transmission of DENVs, and that DENVs demonstrate cross-reactive immunity with ZIKV, the improved specificity of this assay provides a necessary resource for accurately monitoring ZIKV transmission in regions with high DENV endemicity. 

Appendix Additional information about study of Zika seroprevalence in the Philippines.
